# Key professional stakeholders roles in promoting older people's autonomy in residential care 

**DOI:** 10.1177/09697330241247321

**Published:** 2024-07-25

**Authors:** Tanja Moilanen, Riitta Suhonen, Mari Kangasniemi

**Affiliations:** Savonia University of Applied Sciences; 8057 University of Turku; Turku University Hospital; 60654 University of Turku; Turku University Hospital

**Keywords:** Chain of care, Delphi method, ethics, older people, promoting autonomy, residential care

## Abstract

**Background:**

Older people’s autonomy is an ethical and legal principle in everyday residential care, but there is a lack of clarity about the roles and responsibilities of the key professional stakeholder groups involved.

**Research objectives:**

This study aimed to identify and define the roles and responsibilities of the key professional stakeholder groups involved in promoting older people’s autonomy in residential care settings.

**Research design:**

We used a Delphi method with two iterative rounds of online group discussions and collected data from experts in older people’s care in Finland in summer 2020. The data were analyzed using deductive-inductive content analysis methods.

**Ethical considerations:**

According to Finnish legislation, this type of research did not need approval from a research ethics committee. Informed consent from the participants was obtained and they were informed about the voluntary nature and confidentiality of the study and their right to withdraw at any time.

**Results:**

Key professional stakeholders had different roles and responsibilities, but their shared, integrated goal was to achieve older people’s autonomy in residential care settings. Their combined roles and responsibilities covered all aspects of promoting older people’s autonomy, from care and service planning and daily decision-making to service structures that included ethical competencies and monitoring. Multipronged, variable, coordinated strategies were required to identify, assess, and promote autonomy at different levels of care.

**Conclusion:**

Key professional stakeholders need to work together to provide an unbroken chain of care that provides older people with autonomy in residential care settings. In future, more knowledge is needed about how to create structures to achieve the shared goal of older people’s autonomy in these settings.

## Introduction

Autonomy is a fundamental value of older people’s care.^1,2^ Older people have perceived autonomy as an inherent part of their everyday life, as it respects their human dignity.^3,4^ Autonomy refers to someone making their own decisions^5,6^ free of outside intervention and interference,^7^ forming the basis of dignity^8^ and quality of life.^5,9,10^ Thus, autonomy signifies the control of decision-making but also the autonomy of execution to carry out and implement personal choices.^7^ That requires individual capacities,^7,11,12^ such as cognitive and psychological abilities, to make decisions and contemplate potential consequences.^13–17^ In addition, to positive autonomy to execute decisions, it includes also negative autonomy to decline or withdraw actions.^7^ In residential care settings, autonomy is related to decisions and execution concern all daily living activities from of older people’s nutrition, rest and sleep, outdoor and social activities, to hygiene.^3^ Autonomy include also decisions regarding the care and services older people receive and care and their care and service planning.^18^ Decreasing capacity,^19,20^ and living in a facility, can threaten, older people’s autonomy in residential care,^21^ as they are more dependent on others^22–24^ and have limited opportunities to make their own choices.^11,25,26^ Thus, conceptual distinction between the direct and delegated autonomy is relevant in residential care setting: older people decide and act as individual, independent agents but also freely authorize and delegate others to their decisions.^3,4,7^

The definition of autonomy,^12,27,28^ as well as its ethical^2^ and legal basis,^29,30^ are well recognized, but it is still common for older people’s autonomy to be violated in daily practice.^31–33^ These violations can include bans and restrictions on letting older people make their own decisions^20,32,34^ and ignorance of patients’ preferences^22,34,35^ and the principles of good care practices.^36^ Nurses’ actions, and inadequate leadership and management, can increase the risk of violating autonomy,^37,38^ including poorly organized work and resources,^38,39^ lack of appropriate instructions,^37^ and problems with collaboration and communication.^37^ Violations can also be due to organizational factors, such as strict care routines,^34,35,40,41^ poor patient safety cultures,^37,40^ and lack of ethical approaches.^38,42^ In addition, a lack of reporting systems^37,43^ and monitoring^42^ of autonomy violations can increase the risk of violations.

Previous studies have shown that care professionals’ competencies and education were crucial when it came to promoting older people’s autonomy in daily care on residential care setting.^37,39,43^ In addition, other key professional stakeholders, such as managers,^37,38^ administrators,^3,38,42^ principals/clients of services, and supervisory authorities, had their own roles and responsibilities^42^ when it came to promoting older people’s autonomy. However, details about how key professional stakeholders actually promote older people’s autonomy have not been provided. The roles and responsibilities of key professional stakeholders need to be clarified to support the provision of ethical, quality care.

## Aim

The aim of this study was to identify, and define, the key professional stakeholder groups and their roles and responsibilities in promoting older people’s autonomy in residential care settings. We wanted to provide new knowledge that could be used to clarify different stakeholder roles and ensure that the right approaches and policies were in place to support older people’s autonomy in residential care settings.

## Method

**Figure 1.**
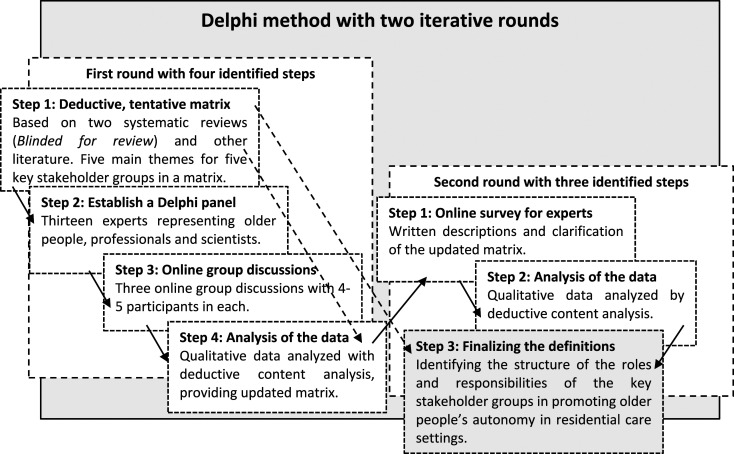
Study design of the two-round iterative Delphi panel study.

We used a Delphi method with two iterative rounds^44–46^ to study older people’s autonomy in residential care settings. The aim of the first round was to identify the roles and responsibilities of different key professional stakeholders and the second round aimed to define consensus. The data were collected in Finland in Summer 2020 and analyzed with deductive-inductive content analysis methods. (Figure 1)

### Round I: Identifying roles and responsibilities

The aim of the first round was to identify the roles and responsibilities of key professional stakeholders involved in promoting older people’s autonomy in residential care. This involved four steps. The first step was to develop a tentative deductive matrix to facilitate expert panel discussions. Two systematic reviews^3,4^ were used to identify the five key professional stakeholder groups and five themes in relation to promoting older people’s autonomy. These were used to construct a discussion matrix. We looked at the choices that older people made and how their close relatives were involved in their care. The matric also covered care practices, the accessibility of the care environment, ethical values and legislation and the resources needed to provide older people with care, including the care workers’ education levels and competencies.

The second step was to establish a Delphi panel (Figure 1), by recruiting experts in the field of older people’s care with the help of key Finnish organizations. These were The Alzheimer Society of Finland, The Union of Health and Social Care Professionals, Centre for Education and Research on Social and Health Services, and a government-funded health and social care organization. The Alzheimer Society is a non-profit organization for the rights of people with dementia and their caregivers. The organizations suggested people who they felt to represent their organizations and would volunteer to be part of the panel. The researcher (TM) sent an invitation letter to them, with information about the study. We also recruited scientists from related disciplines. The thirteen members of the expert panel were two workers from the Alzheimer Society, three healthcare professionals, three scientists from nursing science, two from ethics, and one each from public health medicine, social sciences, and jurisprudence.

The third step was to hold three online group discussions, each comprising 4–5 participants (Figure 1). The researcher (TM) presented the aim of the study and asked the participants to discuss the contents of the tentative matrix that had been developed. The experts were encouraged to criticize, add, remove, and modify the themes and to suggest the responsibilities for all the key stakeholder groups. During the group discussion, the researcher recognized comments by all panelists, filled and made changes to the matrix, and based on the group’s advice and agreement, formulated comments to words, clauses, and sentences in the matrix.^46^

The fourth step was to analyze the data produced by the group discussions, following the principles of deductive-inductive content analysis^47^ (Figure 1). During the discussions, each of the three expert groups produced a matrix that noted the responsibilities of the various key professional stakeholder groups involved in older people’s autonomy and how they could promote older people’s autonomy. We grouped the 408 original notes by the group deductively, according to the various key professional stakeholder groups in the matrix, and then grouped the notes inductively within the groupings, based on their similarities and differences. The notes were condensed and agreed expressions describing the roles and responsibilities of the key professional stakeholders. After that, we removed the overlapping notes and condensed them with the help of previous literature.

### Round II: Definition of the roles and responsibilities

The aim of the second round of the Delphi panel was to define the roles and responsibilities of key professional stakeholders involved in promoting older people’s autonomy in a residential care setting, with the help of the expert panels. These were synthesized into three steps. As a first step, we carried out an online data collection with the nine same experts that took part in the first round (Figure 1). We sent a modified matrix to all the panelists by email, and they were asked to provide written comments and clarify the responsibilities of the key professional stakeholder groups that had been identified.

The second step was to analyze the second-round data that we received (Figure 1). The nine panelists provided 236 written comments and notes and we removed two unclear notes. The comments and notes were words, clauses, or sentences, and they concerned the content and expressions of the matrix, and importance of the responsibilities of different key professional stakeholders. We analyzed the content deductively based on their similarities and differences and grouped 178 notes below the different stakeholders.

**Figure 2.**
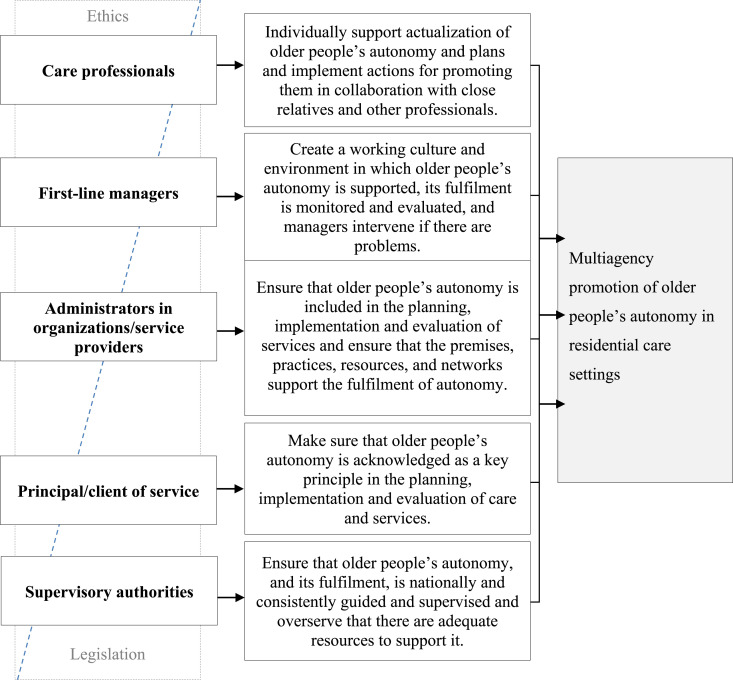
Main roles and responsibilities of key stakeholder groups promoting older people’s autonomy in residential care settings.

**Figure 3.**
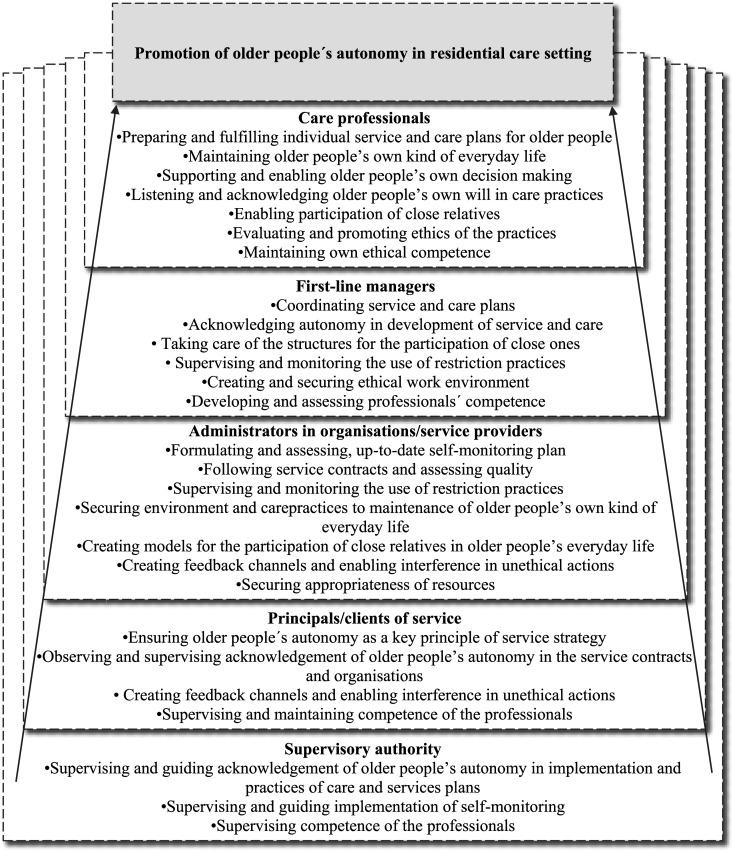
The roles and responsibilities of key stakeholder groups to promote of older people’s autonomy in residential care setting.

The third and final step (Figure 1) was to formulate and finalize the definition of the roles and responsibilities of the key professional stakeholder groups promoting older people’s autonomy in residential care settings (Figure 2 and 3).

### Ethical considerations

We followed ethical principles throughout the study process.^48^ According to Finnish legislation, this type of research did not need approval from a research ethics committee.^49^ We obtained informed consent from the participants and they were informed about the voluntary nature and confidentiality of the study and their right to withdraw at any time.^50^ Although the traditional Delphi method emphasizes anonymity between participants,^44,51^ we felt that the shared discussions with experts would produce a wider and more multidimensional understanding about the topic. Thus, the participants were not anonymous, but the confidentiality of all discussions was stressed and agreed. We illustrated the abstraction process of the data with straight quotations, but to ensure the anonymity of participants, we have not mentioned their organizational positions in the quotations.

## Results

The key professional stakeholder groups who promoted older people’s autonomy in residential care settings were care professionals, first-line managers, service provider administrators, those responsible for organizing services and national supervisory authorities. Care professionals referred to all health and social care staff involved in older people’s daily care, regardless of their educational background. First-line managers referred to the people in charge of daily practice in care homes. Organizations and service providers referred to private or public stakeholders providing the services. Principals/clients of services referred to municipalities and other stakeholders responsible for providing care services for older people. Supervisory authorities referred to responsible officers by the state. All stakeholders had their own roles, based on ethics and legislation (Figure 2), with intertwined responsibilities (Figure 3).

### Care professionals support older people’s autonomy during everyday care

The role of care professionals is to support older people to achieve their individual autonomy (Figure 2). They do this by planning and implementing supporting care practices in collaboration with the older people’s close relatives and other professionals. Care professionals are responsible for considering an older person’s autonomy when they prepare and fulfill their individualized care and service plans. These care practices include supporting and enabling older people to make their own decisions and maintain their individual everyday life, while acknowledging their wishes. Enabling their close relatives to get involved in older people’s lives has also been recognized as supporting good practice. In addition, the professional’s role is to evaluate and promote practices from an ethical point of view and maintain their own ethical competencies in older people’s care (Figure 3).

“In particular, the individual professional is responsible for a respectful attitude and the development of professional skills to recognize autonomy.”

### First-line managers create care environments that emphasize autonomy

The responsibility of first-line managers in residential care is to create and maintain working cultures and environments that support older people’s autonomy (Figure 2). This can take the form of coordinating care and service plans that recognize older people’s autonomy and providing close relatives with opportunities to get involved. In addition, first-line managers are responsible for monitoring and preventing restrictions, facilitating non-restrictive care practices and creating structures that enable close relatives to get involved in older people’s care. Their role is also to create a secure work environment and to develop, and maintain, the ethical competencies of care professionals so that care professionals can support and acknowledge older people’s autonomy in residential care (Figure 3).

“The employer must ensure that there is an adequate amount of trained nursing staff so that there is time and opportunities for genuine encounters [with older people].”

“It is necessary to assess the ratio of human resources to the competencies of the staff: the number of staff does not guarantee “automatically” the realization of autonomy.”

### Administrators in organizations and service providers ensure that autonomy is acknowledged in residential care

The role of the administrators working for organizations and service providers is to ensure that the planning, implementation, and evaluation of the older people’s care services include older people’s autonomy. They also ensure that the resources are available so that older people’s autonomy is fulfilled (Figure 2). Their task, at an organizational level, is to formulate and carry out a regulated self-monitoring plan that acknowledges older people’s autonomy, but still meets service contracts and focuses on quality services. The administrator’s role is also to supervise and monitor non-restrictive practices and make sure that secure care environments and care practices make it possible to support older people’s autonomy in collaboration with their close relatives. They are responsible for creating feedback channels that enable clients, the general public and staff to report possible unethical actions in care. In addition, they support managers who need to step in if anything unethical happens. They also need to ensure that there are sufficient resources and that these are used appropriately to enable the organization to provide safe, quality care (Figure 3).

“The service provider is obliged to comply with the legislation, but there are a lot of things that can be done on top of it that improve the quality of services. The unit must have a self-monitoring plan according to law.”

### Principals/clients of services ensure that older people’s autonomy forms the basis of care and services

Principals/clients of services are responsible for ensuring that older people’s autonomy is the key principle that drives the planning, implementation, and evaluation of care services in residential care (Figure 2). Making sure that service contracts underline the importance of observing and supervising older people’s autonomy, and that these form part of the practices of the organizations, are a way of promoting autonomy. In addition, principals/clients of services are responsible for ensuring that feedback channels are available and that any potential unethical actions by professionals are recognized and stopped. They are also responsible for making sure that care workers have the up-to-date ethical competencies they need (Figure 3).

“[Principals/clients of services have] responsibility to ensure the legislative and care-related starting points for autonomy and their implementation in service planning, tendering, during the contract period and at the end of the service.”

### Supervisory authorities create national consistent and unified support for autonomy and monitoring

The supervisory authorities are responsible for making sure that older people can achieve autonomy, by providing national guidance and supervision and making sure that adequate resources are available (Figure 2). They need to ensure that service structures support older people with different service needs. In addition, they need to supervise and guide the implementation of self-monitoring practices that ensure that older people achieve autonomy in residential care settings and ensure that the professionals that care for them are competent (Figure 3).

“Although supervision and guidance cannot ensure that the service system meets the needs of the older people and their autonomy with sufficient resources – it can monitor that the services are implemented with sufficient resources.”

## Discussion

Identifying, assessing, and promoting older people’s autonomy requires multipronged and variable strategies at different levels of care,^42^ including individual professionals, organizations, and supervisory authorities. According to our results, all key professional stakeholders have their own roles and responsibilities, but their shared, integrated goal is to achieve older people’s autonomy in residential care settings. Collaboration across professional and authority boundaries is needed to achieve these shared goals and to prevent older people’s autonomy from being violated in residential care settings.

Providing opportunities for older people’s perceived autonomy in residential care settings enables older people to make daily decisions about their life. For example, a number of factors need to come together to enable older people to make decisions about their eating^14,18,38^ or rest habits. The care workers need the competencies to identify autonomy as a principle in care and^14,52^ the first-line managers need to coordinate care and professional competencies.^53^ The administrators and organizations and service level facilitation also need to play their roles.^3,38^ Stakeholders are needed at all levels in order to complete the chain. Care professionals need to promote older people’s autonomy in every nursing care action. First-line managers need to create care environments that emphasize autonomy throughout residential care settings. Principals/clients of services need to build services based on older people’s autonomy. Supervisory authorities need to create consistent and unified support and monitoring for older people’s autonomy in residential care settings. Despite this, care workers have reported that they have had to compensate for incomplete management or resources.^38,39,54,55^ In addition, supervisory monitoring, without advice or consequences, has been led to frustration in organizations.^56^ Collaboration across professional and authority boundaries is needed to ensure that there are no gaps in the chain. Elaborating different roles and responsibilities creates a basis for discussions and identifies developmental needs for care practices. It is crucial that all key professional stakeholders are aware of their different roles and responsibilities and can concentrate on fulfilling their own duties. In addition, more research is needed on strategies to develop mutual collaboration with local and national authorities.

The current challenge is to create an unbroken chain to promote older people’s autonomy in residential care settings that is connected to all levels of key professional stakeholders. On the care professionals’ level, the challenge is linked to increased workload with decreased human resources. There is a risk that the educational level of care workers will be reduced, or even removed, to meet workforce demands, and that the turnover of all professional groups will increase.^57^ This poses a serious challenge for first-line management level to ensure professionals’ ethical competencies and their abilities to support older peoples’ autonomy. On the administrators and service providers’ level the challenges to ensure unbroken chain is connected to the increased variation on service providers. For example, privatization in Finland has over-intensified services, compromised organizational resources, and challenged staffing levels^57,58^—possessing older people’s autonomy on the secondary position. Finally, the changes in the structure of service provision challenge the supervisory authorities to ensure the clear and visible regulations and structures that enable older people to achieve autonomy in residential care settings. All the levels of key professional stakeholders need working conditions that enable the service chain to provide ethically safe care for older people.^42^ More research is needed about the factors that could affect the development and execution of care chains.

Finally, the primary question is about the rights of older people to receive respectful and good-quality care.^59^ Autonomy is a fundamental human and patient right and the violation of autonomy is usually connected to the violation of other ethical principles as well, such as integrity or equality.^2^ It is obvious that older people are the most important stakeholders^60^ in their care. However, achieving autonomy requires resources and that should not discriminate against older people.^61,62^ Care structures and services should ensure that older people have the same circumstances and opportunities to achieve autonomy during their time in residential care settings. Thus, in the future, there is need for more knowledge on two crucial issues: how older people and their close relatives evaluate the roles and responsibilities of key professional stakeholders to promote older people’s autonomy in the residential care setting, and how older people and their close relatives see their own role on the chain.

### Strengths and limitations

The Delphi method proved to an effective way to identify key practices and study ethical issues.^44,46^ As a strength of the study, we developed the Delphi study design by carrying out literature reviews, using information from the relevant papers we identified and organizing two rounds of the expert panel. The limitations focus on the number of the participants and their different views on the topic: the low number of participants is the risk to limited knowledge, but the strength of our data collection is that it provided saturated data.^44^ In addition, as the aim of the Delphi method was to achieve consensus, the participants were encouraged to formulate notes in agreement, and thus, we were not able to report the participants’ different views on the topic.^45^ However, reporting different participants individual viewpoints would have provide understanding of the differences of approaches. During the group discussion, the researcher ensured that all participants had an opportunity to express their comments and in case of unclear expressions, the researcher encouraged the group reflect more to achieve consensus. The experts provided wide experience on the topic from various perspectives, as the Delphi panels included representatives of older people, care professionals, first-line managers, and scientists. The workers by the Finnish Alzheimer Society were representing of diverse group of older people. However, the wider involvement of older people with various backgrounds would have been deepened understanding about the key professional stakeholders’ roles in promoting older people’s autonomy in residential care settings.

## Conclusion

One of the basic rights that citizens have is that they can benefit from autonomy if they live in residential care when they grow older. Thus, service users’ autonomy is not isolated from care and service structures and long-term planning is needed to achieve it, with input from all levels of key professional stakeholders. All key professional stakeholders need to be aware of their own and the other stakeholders’ roles in the chain and have the intention to develop structures and collaboration. It is crucial that key professional stakeholders establish how older people perceive their autonomy when they evaluate how autonomy is executed in residential care settings. However, in the future, more knowledge is needed about the roles and responsibilities of older people and their close relatives on the promotion of autonomy in the residential care service setting. In addition, knowledge is more knowledge is needed about how to create and evaluate success of the care chains that achieve the shared goal of older people’s autonomy in these settings.
